# Recent Advances in Non-Invasive Blood Markers for Intraductal Papillary Mucinous Neoplasm Grading

**DOI:** 10.32604/or.2026.084411

**Published:** 2026-08-13

**Authors:** Liang Chen, Yitong Yuchi, Qian Zhu

**Affiliations:** 1 Second Clinical College, Wuhan University, Wuhan, China; 2 School of Basic Medical Sciences, Wuhan University, Wuhan, China; 3 Department of Hepatobiliary and Pancreatic Surgery, Zhongnan Hospital of Wuhan University, Wuhan, China

**Keywords:** Intraductal papillary mucinous neoplasm (IPMN), non-invasive grading, blood biomarkers, serum proteins, inflammatory indicators, circulating free DNA (cfDNA), MicroRNA (miRNA), telomere, clinical translation

## Abstract

Intraductal Papillary Mucinous Neoplasm (IPMN) is a major precancerous lesion of pancreatic ductal adenocarcinoma. Accurate risk stratification of IPMN is key to preventing and controlling pancreatic cancer. At present, clinicians mainly grade IPMN following the Kyoto consensus guidelines, together with imaging examinations and traditional serum biomarkers like CA19-9 and CEA. This review unveils the latest research progress of non-invasive blood biomarkers for the grading of IPMN, including the diagnostic performance, molecular mechanisms, and current clinical translation of several types of markers. ApoAII has already been applied in clinical practice, and miRNA combinations, circulating cell-free DNA (cfDNA) methylation panels, and other markers show excellent value in IPMN stratification. However, the field still faces bottlenecks in clinical translation. In the future, researchers should focus on exploring specific targets for IPMN malignant transformation, unifying detection standards, and building multi-dimensional combined evaluation models. These efforts will help promote the application of non-invasive blood biomarkers in accurate IPMN grading and support the early screening and treatment of pancreatic cancer. It not only enables doctors to comprehensively understand the research status of existing non-invasive blood markers for IPMN, but also provides guidance for exploring promising non-invasive biomarkers in future studies.

## 1 Introduction

Intraductal Papillary Mucinous Neoplasms (IPMN) start in the main pancreatic duct or its major branches and secrete a large amount of mucus. They are the most common pancreatic cystic lesions, accounting for more than 40% of all pancreatic cystic tumors [1].

In studies published after 2020, population epidemiological data demonstrate that the prevalence of pancreatic cystic lesions and intraductal papillary mucinous neoplasms (IPMNs) rises sharply with age. Among individuals aged over 80 years, the prevalence of pancreatic cysts reaches up to 75.7%, and approximately half of these cystic lesions are IPMNs. From 2010 to 2017, the incidence of IPMNs in the United States showed a continuous increase. Meanwhile, the annual progression rate of existing lesions was 0.47%. For branch duct IPMNs (BD-IPMNs), the cumulative risks of pancreatic cancer are 6.6% and 12% at 10 and 15 years of follow-up, respectively. Even BD-IPMNs remaining stable for 5 years still carry a certain malignant transformation risk, whereas the additional cancer risk is not statistically significant in elderly patients over 65 years with stable lesions. Current clinical guidelines lead to an over-treatment rate of approximately 75% for IPMN resections. Novel biomarkers derived from cyst fluid and genetic testing can substantially improve the differentiation between benign and high-risk IPMNs [2].

**Figure 1:**
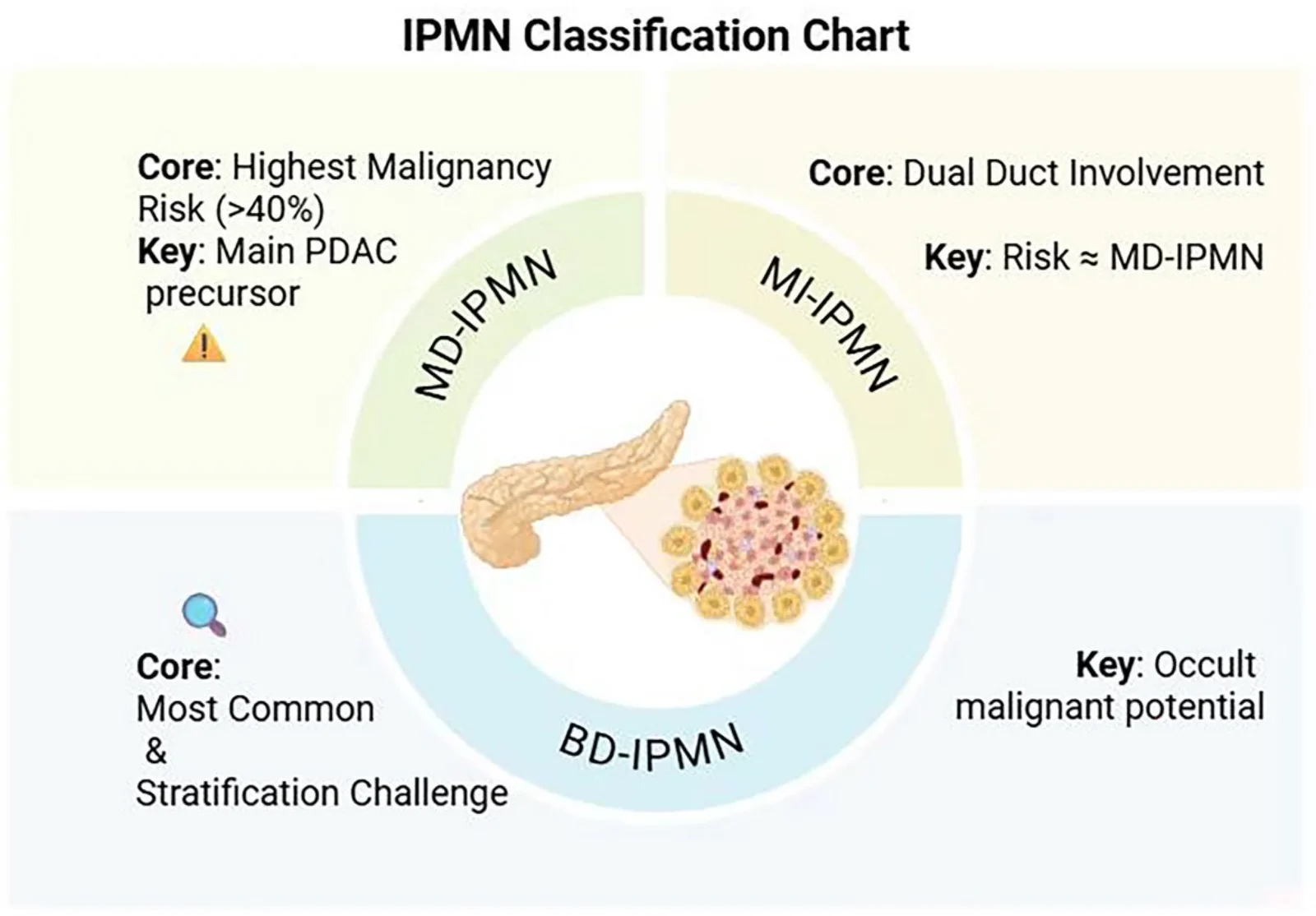
IPMN Classification Chart. This diagram illustrates the three major subtypes of IPMN, along with their core clinical features. Main-duct IPMN (MD-IPMN) involves the main pancreatic duct, represents the main precursor lesion to pancreatic ductal adenocarcinoma (PDAC), and carries the highest malignancy risk (>40%). Mixed-type IPMN (MI-IPMN) is characterized by dual duct involvement of both the main and branch ducts, with a malignant risk comparable to that of MD-IPMN. Branch-duct IPMN (BD-IPMN), the most common clinical subtype involving only secondary pancreatic ducts, poses the primary challenge for risk stratification due to its inherent heterogeneity and the presence of occult malignant potential in a subset of cases.

IPMN are divided into three types according to which part of the pancreatic duct system is affected. In Fig. 1, we sorted out the features of various IPMN subtypes. They are main duct-type (MD-IPMN), branch duct-type (BD-IPMN) and mixed-type (MI-IPMN). MD-IPMN lesions are usually local and only in the large pancreatic ducts. However, diffuse dilatation of the Wirsung duct can be observed in certain patients. BD-IPMN arises exclusively within the small pancreatic ducts and predominantly locates in the pancreatic head. Subtypes of IPMN differ significantly in malignant transformation risk and incidence. BD-IPMN is the most prevalent subtype, whereas MD-IPMN has the highest risk of malignant transformation [1].

IPMN have attracted a lot of clinical attention in recent years. First, the incidence of IPMN has been increasing year by year. Second, IPMN are one of the most common precancerous lesions of pancreatic ductal adenocarcinoma (PDAC). PDAC is the most common malignant pancreatic tumor, and it is also one of the deadliest cancers. Its mortality rate is almost the same as its incidence rate. The 5-year survival rate after diagnosis is only 13% [3]. This bad situation is related to two characteristics of PDAC. One is the lack of specific markers, and the other is the poor response to chemotherapy. Surgeons want to prevent PDAC and other malignant changes by monitoring the tumor development in IPMN patients.

Risk stratification is the main work of IPMN monitoring. Patients with Low-Grade Dysplasia IPMN (LGD-IPMN) can receive regular follow-up checks. Patients with high-grade dysplasia IPMN (HGD-IPMN) need surgical resection. At present, the risk stratification of IPMN mainly relies on the Kyoto Guidelines, which were formerly the Fukuoka Guidelines. The guidelines divide IPMN into three grades: high-risk stigmata (HRS), worrisome features and no specific features. Doctors evaluate IPMN by combining imaging markers with serum CA19-9 and cyst fluid CEA. The imaging markers include main pancreatic duct dilation and cyst size [1]. But this evaluation method has great limitations. It is very likely to cause misdiagnosis and missed diagnosis. It leads to two problems: unnecessary surgery and missed detection of early lesions. These problems harm patients’ health and increase economic costs [3,4,5].

To improve the accuracy of IPMN risk stratification, researchers are trying to find more effective multi-omics markers and set up more complete evaluation standards. They also hope to realize non-invasive IPMN risk assessment through serological testing. With the breakthroughs in multi-omics technology, tumor microenvironment research and non-invasive detection technology, researchers can do two things at the same time. They can find markers with cross-dimensional technologies, and study the micro mechanisms of IPMN tumors. They can also explore the ways to apply these research results in clinical practice.

This review categorizes and summarizes the latest non-invasive grading biomarkers for IPMN in the following sections, including miRNA, inflammatory markers and other related indicators. It comprehensively evaluates these markers from multiple dimensions such as diagnostic performance and detection difficulty, and finally objectively assesses their prospects for clinical application. This study intends to deepen readers’ understanding of non-invasive blood grading biomarkers for IPMN, and provide instructive suggestions for future relevant research and clinical translation.

## 2 Grading Criteria for IPMN

### 2.1 Pathological Grading

The 2019 WHO criteria classify IPMN into two grades based on cellular atypia, structural atypia and invasive status.

Low-grade dysplasia (LGD) encompasses IPMN adenomas and borderline IPMN. The cells show mild to moderate atypia, and they are arranged in a single layer with simple structures. Some cases are between LGD and HGD. LGD carries a relatively low risk of malignant transformation. It has mostly served as the “benign control” group in studies within the preceding five-year period. High-grade dysplasia (HGD) refers to IPMN with carcinoma *in situ*. The cells have obvious atypia, and they are arranged in a pseudostratified layer with complex structures. HGD is a key precancerous stage of pancreatic cancer. The risk of becoming malignant is as high as 30%–50%. It is also a major target for non-invasive grading. Any lesion showing definitive stromal invasion qualifies as invasive carcinoma (IC). Clinically, doctors need to intervene according to the diagnosis and treatment standards for pancreatic cancer. IC is the “extremely high-risk type” that needs to be identified as a priority in non-invasive grading [1,6].

Compared with the 4th edition of the WHO criteria in 2010, the 2019 update merged LGD-IPMN and Moderate-Grade Dysplasia IPMN (MGD-IPMN) to simplify clinical management. Some medical institutions even use a two-level standard of high-risk and low-risk for the pathological grading of IPMN [7]. But this grading standard frequently results in misdiagnosis and inappropriate surgical decisions, inflicting unnecessary economic burdens and health detriments on patients.

### 2.2 Clinical Risk Stratification

There are many international standards for IPMN risk stratification for reference. For example, the International Association of Pancreatology (IAP) Kyoto Guidelines, European Society of Gastrointestinal Endoscopy (ESGE) Guidelines and American Gastroenterological Association (AGA) Guidelines. This review is mainly sorted out according to the latest 2024 Kyoto Guidelines of the International Association of Pancreatology (IAP) [8].

In general, the core of IPMN risk stratification employs a two-layer evaluation framework of high-risk stigmata (HRS) and worrisome features (WF). This framework functions to guide surgical decisions and follow-up strategies. HRS draws on four indicators: obstructive jaundice related to pancreatic head cystic lesions, enhancing mural nodules ≥ 5 mm or solid components in the cyst, main pancreatic duct (MPD) diameter ≥ 10 mm, and suspicious or positive cytological test results. HRS highly suggests the risk of malignant transformation, which means HGD or IC. It is recommended to conduct a surgical evaluation for such patients. Notably, compared with previous guidelines, the 2024 IAP Kyoto Guidelines officially incorporate suspicious or positive cyst fluid cytology into HRS as the fourth high-risk indicator.

WF is based on several indicators. They are cyst diameter ≥ 30 mm, enhancing mural nodules less than 5 mm, MPD diameter of 5–9 mm, cyst wall thickening or enhancement, abrupt change in MPD lumen with distal pancreatic atrophy, new-onset diabetes or elevated CA19-9 (more than 37 U/mL), rapid tumor growth ≥ 2.5 mm per year, and a history of tumor-related acute pancreatitis. The more WF indicators a patient has, the higher the risk is. The risks with 1, 2 and 3 WF indicators are 22%, 34% and 59% respectively. The risk reaches 100% with 4 or more WF indicators. It is recommended to conduct further EUS evaluation for such patients. If a patient has no HRS or WF indicators, with a cyst less than 30 mm and a main pancreatic duct less than 5 mm, it is a low-risk feature. Routine follow-up and monitoring are enough for such patients [8,9].

Different subtypes of IPMN are also important bases for risk assessment. According to anatomical classification, MD-IPMN has an extremely high risk of becoming malignant (more than 40%), so surgical resection is mostly recommended. BD-IPMN has an overall low malignant risk, but it is the main difficulty in risk stratification. MI-IPMN has a malignant risk between MD-IPMN and BD-IPMN. But the Kyoto Guidelines recommend that its malignant transformation risk is similar to that of MD-IPMN, so the same management method should be adopted [8].

### 2.3 Latest Advances in Traditional Biomarkers CA19-9 and CEA

CA19-9 and CEA remain the most commonly applied conventional serum biomarkers for risk stratification and grading of intraductal papillary mucinous neoplasms (IPMN) in routine clinical settings [10,11,12]. Qian et al. [13]’s meta-analysis validated the moderate diagnostic performance of both markers for distinguishing malignant or invasive IPMN from benign lesions.

However, their inherent limitations severely restrict clinical reliability. Yang et al. [11] demonstrated that CA19-9 is strongly associated with invasive carcinoma but not high-grade dysplasia, with frequent false positives in cholestasis and false negatives in Lewis-negative individuals. Perri et al. [14] further highlighted that conventional thresholds yield low accuracy for early malignant progression. For CEA, Wang et al. [10] reported a consistently high specificity of 93–95% but a strikingly low sensitivity of only 18–28%, rendering it ineffective for screening. Current evidence suggests that such low sensitivity makes both markers unreliable as standalone tests and introduces substantial uncertainty in clinical decision-making.

Recent improvements focus on optimized thresholds and combinatorial panels. Qian et al. [13] proposed revised cutoff values for CA19-9 and CEA that slightly enhance stratification. Servin-Rojas et al. [15] emphasized that CA19-9 > 100 U/mL serves as a strong predictor of invasive disease. Notably, Qian et al. [13] also showed that combining CA19-9 with CA125 significantly improves detection sensitivity, particularly in CA19-9-negative cases. From a practical standpoint, these strategies are low-cost and easy to implement, yet their overall ability to reduce unnecessary surgery remains modest.

In summary, while CA19-9 and CEA are widely accessible, we regard their diagnostic credibility as limited and their long-term potential as constrained by fundamental biological drawbacks. They can be used only as adjunctive tools, and more specific biomarkers are urgently needed for reliable IPMN grading.

### 2.4 Optimization of Grading Criteria and Driving Factors for Non-Invasive Detection in the past 5 Years

In recent years, to address the potential issues caused by merging MGD and LGD mentioned above, the Kyoto Guidelines have refined the pathological diagnostic threshold of MGD. The threshold is a cellular atypia ratio more than or equal to 10% and less than 50%, and a glandular structural disorder that does not meet the HGD standard. This helps reduce the misdiagnosis and erroneous judgment between LGD and HGD, and improves the consistency of grading. At the same time, on the basis of previous guidelines, a new grading definition of “microinvasion” is added, with an invasive depth of less than 5 mm. It includes the early stage of IC, and is connected with the diagnostic criteria of “microinvasive IPMN” in previous guidelines, which serves as a valuable adjunct to preoperative risk assessment [8]. The 2023 European Society for Medical Oncology (ESMO) Guidelines and the 2025 Guidelines of the Chinese Society of Gastroenterology both refer to the Kyoto Guidelines. They all emphasize the dichotomous grading of “LGD/MGD vs. HGD/IC”, and realize the unification among different guidelines [16].

With the popularization of the concepts of “precision medicine” and “minimally invasive diagnosis and treatment”, as well as the vigorous promotion of various guidelines, non-invasive grading has gradually replaced invasive pathological biopsy as the preferred preoperative assessment method. In the risk stratification of IPMN, serological testing is the core traditional method for non-invasive initial screening. Traditionally, clinicians have utilized CA19-9 as the core marker for serological testing, and serum CEA as the secondary marker. This method realizes the first-line non-invasive, convenient and cost-effective risk stratification of IPMN. Given its suboptimal diagnostic accuracy, it cannot be used as a standalone tool for IPMN risk stratification [17]. With the improvement of people’s requirements for health and quality of life, highly accurate non-invasive blood markers have become a research hotspot. People need efficient and accurate non-invasive blood markers to improve the prognosis of IPMN patients. These markers can reduce unnecessary surgical operations and lower medical costs. They can also improve the efficiency of IPMN diagnosis and treatment, and meet the needs of rational allocation of clinical medical resources.

## 3 New Progress in Core Blood Markers in the past 5 Years

**Figure 2:**
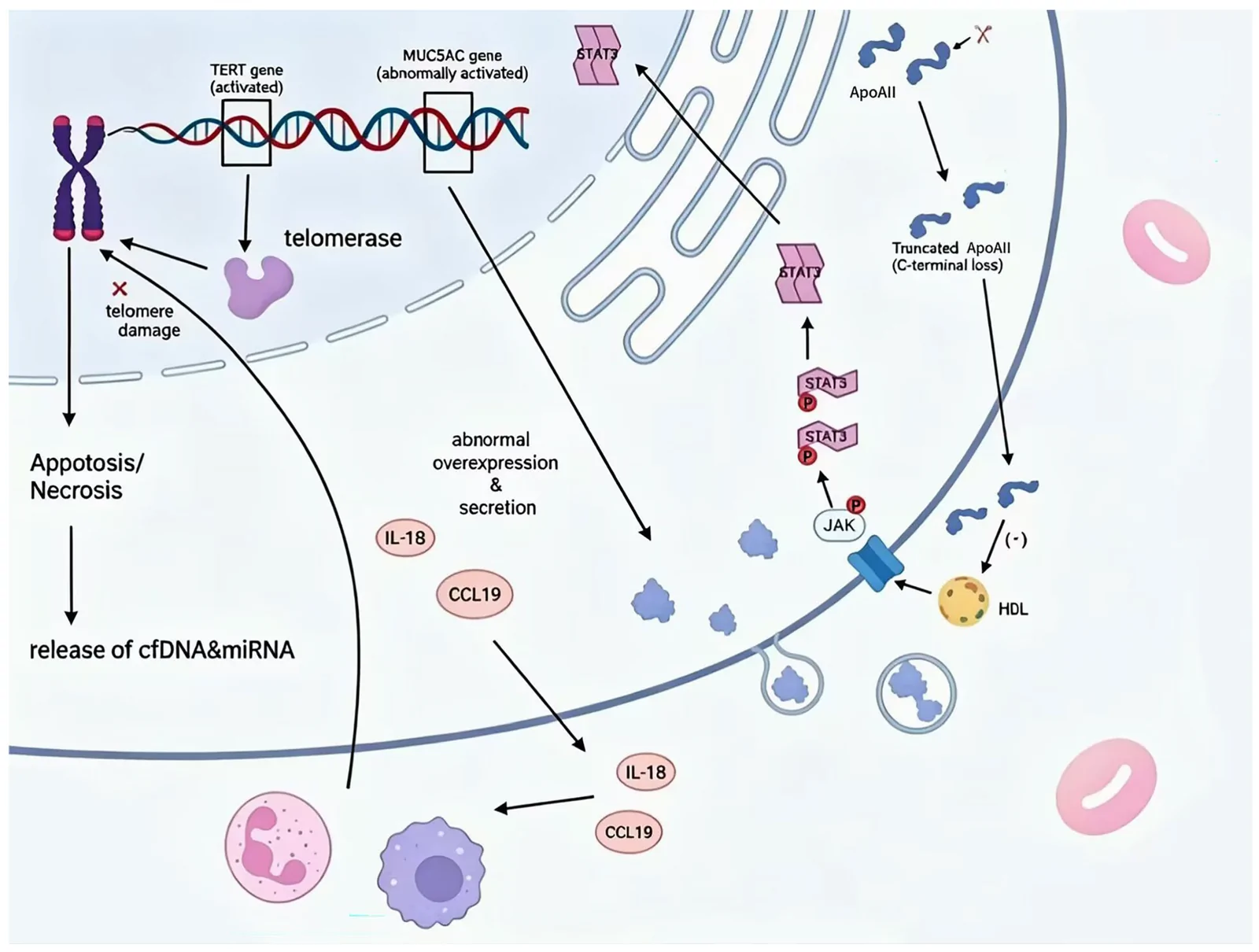
Schematic representation of the molecular mechanisms underlying blood-based biomarkers associated with malignant progression in IPMN. This diagram illustrates the key molecular events during IPMN progression and their regulatory pathways that produce measurable peripheral blood biomarkers. Telomere dysfunction triggers compensatory TERT upregulation and telomerase activation; irreversible telomere damage induces apoptosis and necrosis, releasing circulating cfDNA and miRNAs. Abnormally secreted IL-18 initiates inflammatory cascades within cystic lesions and triggers robust CCL19 upregulation and secretion; the secreted CCL19 serves as a downstream effector molecule to activate the Janus Kinase/Signal Transducer and Activator of Transcription 3 (JAK/STAT3) signaling cascade, while IL-18 can directly stimulate this pathway in an independent manner, jointly constructing a sustained pro-inflammatory signaling loop that disrupts immune homeostasis and accelerates malignant transformation of epithelial cells. Meanwhile, ApoAII is cleaved to form truncated ApoAII with C-terminal loss, which inhibits High-Density Lipoprotein (HDL) function.

We reviewed the hottest research areas in the past five years including EVs and miRNAs, and elaborated on novel blood biomarkers for IPMN risk stratification in terms of diagnostic performance, clinical translation and future application prospects. The functional mechanisms of individual biomarkers are illustrated in Fig. 2 for better comprehension.

### 3.1 Serum Protein Markers

#### 3.1.1 Extracellular Vesicles (EVs)

Extracellular vesicles (EVs) are a family of heterogeneous membrane structures released by cells. They are membranous vesicles released by all cell types, which differ from classical secretory vesicles. Their core function is to mediate intercellular communication. By enabling the transfer of bioactive molecules including proteins, lipids, and nucleic acids, EVs participate in a wide spectrum of physiological and pathological processes [18]. Previously, many studies have taken EVs as liquid biopsy markers for cancer. The research mainly focuses on four major cancers: prostate cancer, pancreatic cancer, lung cancer and breast cancer [19].

A previous preclinical study has demonstrated the predictive value of the MUC5AC marker in PDAC, yet this finding has not been validated by rigorous large-scale studies [20]. Recently, Yang and his colleagues developed and applied the Digital Extracellular Vesicle Screening Technology (DEST) in an innovative way. They used it to analyze EV biomarkers in plasma, and identified MUC5AC as a specific biomarker to distinguish invasive IPMN [11]. MUC5AC is a secretory mucin gene. It encodes high-molecular-weight glycoproteins, which are mainly expressed in the surface epithelial cells of the gastric mucosa. These glycoproteins form a mucus layer to protect the gastric mucosa from erosion by gastric acid and digestive enzymes. As MUC5AC is not expressed in the normal pancreatic duct, it represents a highly potential marker for identifying malignant transformation of pancreatic duct tissue [21].

The DEST they developed can detect EVs with high efficiency. Detection can be completed within 2 h, requiring only 1–10 μL of plasma. Verification shows that MUC5AC as a marker found by this method has a much better diagnostic effect than the traditional serological markers CA19-9 and CEA. Its sensitivity, specificity and diagnostic accuracy for invasive IPMN are 100%, 82% and 96% respectively. When MUC5AC is combined with imaging and HRS detection, the Area Under the Receiver Operating Characteristic curve (AUC) rises to more than 0.83, and the AUC for invasive IPMN reaches 1.0. In addition, the diagnostic performance of other extracellular vesicle biomarkers including MUC1, MUC2 and MUC4 etc. for risk stratification of IPMN was also evaluated in this study. Ultimately, 16 biomarkers capable of generating stable antibody pairing for detection were identified, among which MUC5AC stood out as the core candidate. Further analyses revealed that Glypican 1 (GPC1) and Thrombospondin 1 (TSP1) exhibited moderate predictive value, alongside a five-protein EV signature panel consisting of MUC1, GPC1, Epidermal Growth Factor Receptor (EGFR), Epithelial Cell Adhesion Molecule (EpCAM) and WNT-2. Nevertheless, all of these candidates and the multi-marker panel yielded inferior overall diagnostic efficacy compared with MUC5AC alone, as well as the combinatorial strategy integrating MUC5AC detection with imaging assessments [11]. Later, the team carried out another related study. They found a combination of four markers: Das-1, Vimentin, Chromogranin A and Carbonic anhydrase IX (CAIX). This combination can effectively distinguish atypical SCA from BD-IPMN that needs surgery, with an AUC of 0.99 [22].

DEST is an ultrasensitive detection technique with near-single-EV resolution, which is essentially an improved magnetic bead-based digital enzyme-linked immunosorbent assay (ELISA). Compared with traditional ELISA, it is 10,000 times more sensitive, requires only a small sample volume, can complete detection in less than 2 h, allows high-throughput processing of clinical samples without ultracentrifugation, and is suitable for routine clinical testing. In addition, it has a relatively low cost and is appropriate for clinical promotion. However, this biopsy method also has obvious shortcomings. At present, relevant studies mainly focus on four major cancers, and there is a lack of universal biomarkers for other cancers and different subtypes of pancreatic tumors. The independent diagnostic ability of a single biomarker is limited, while DEST has not yet achieved clinical standardization. The heterogeneity of EVs may also affect detection stability, making clinical translation rather difficult. Therefore, in the future, we need to further expand the coverage of EV-specific biomarkers across cancer types and subtypes, optimize the multi-marker combined detection model, improve the standardized clinical procedure of DEST to solve the problem of detection stability, and deepen the multimodal combined diagnosis of EV markers with imaging and clinical indicators, so as to enhance their independent diagnostic value and accelerate clinical translation and popularization.

#### 3.1.2 Apolipoprotein A-II (ApoAII)

Apolipoprotein A-II (ApoAII) is the second major component of HDL and mediates lipid transport and metabolism. It forms dimers to stabilize HDL and has three isoforms: heavy untruncated homodimer ApoAII-1 (ApoAII-ATQ/ATQ), semi-truncated heterodimer ApoAII-2 (ApoAII-ATQ/AT), and fully truncated homodimer ApoAII-3 (ApoAII-AT/AT). Two additional deeply cleaved variants (ApoAII-4 and ApoAII-5) with further C-terminal amino acid loss were also newly identified in pancreatic cancer plasma samples in the same study. [23,24,25,26]. Its key abnormality is C-terminal truncation, which disrupts isoform balance, impairs HDL function, activates pro-tumor pathways, and promotes cancer progression [24,25].

Kazufumi Honda’s team cooperated with 8 medical institutions in Japan and Germany. They analyzed 1314 plasma/serum samples and found that the half-truncated ApoAII (17,252 m/z) decreased significantly in pancreatic cancer patients (*p* = 1.36 × 10^−21^). Its AUC for the diagnosis of stage I pancreatic cancer reaches 0.868, which is better than the traditional marker CA19-9 (AUC = 0.774). The study also confirmed that the combined detection of ApoAII-ATQ/AT and non-glycosylated ApoCIII-0 (8766 m/z) can raise the AUC for pancreatic cancer diagnosis to 0.903 [24]. Klaus Felix’s team studied 305 IPMN patients. They found that the serum level of ApoAII-ATQ/AT decreased significantly in patients with HGD and IPMN-associated cancer. The diagnostic Areas Under the Curves (AUCs) were 0.910 and greater than 0.940, with sensitivities of 70.6% and 83.3% (specificity 94.4%). These performance metrics are much superior to those of CA19-9 [26]. Hasegawa et al. [23] analyzed the plasma of 212 IPMN patients. They found that the ApoAII-i index decreased significantly in patients with HRS/WF. Its AUC for distinguishing high-risk IPMN is 0.676, which is significantly better than CA19-9 (AUC = 0.554, *p* = 0.029).

In addition, all existing studies show that the abnormality of ApoAII in plasma/serum samples is closely related to the malignant transformation process of IPMN. However, no significant difference in the ApoAII modification pattern was found among IPMN patients with different degrees of dysplasia in pancreatic juice samples. This suggests that the choice of sample type is critical for the clinical value of ApoAII detection [26]. More clinically significant, a prospective study based on the European Prospective Investigation into Cancer and Nutrition (EPIC) cohort shows that the combined detection of ApoAII-ATQ/AT and CA19-9 can significantly improve the early warning ability of pancreatic cancer. In the time window of more than 6–18 months before diagnosis, the C-statistic of the combined detection reaches 0.74 (0.71 for CA19-9 alone, *p* = 0.022). The sensitivity reaches 36% at 98% specificity (29% for CA19-9 alone). It provides a longer early warning window for the early screening of pancreatic cancer [27]. However, another study demonstrated that this combination could not significantly improve the ability of ApoAII in risk stratification of IPMN [23].

Besides, Olga Gursky and others identified two amyloidogenic hotspots in ApoAII (residues 10–18 and 60–70) through structural biology analysis. C-terminal extension mutations in patients with hereditary amyloidosis will introduce an additional hotspot (residues 79–87). These hotspots work together to drive the conformational transition from α-helix to β-sheet and the formation of insoluble fibrils [28]. Felix K and others analyzed IPMN tissue samples. They found that somatic truncation modifications of ApoAII (including ApoAII-5) are related to the invasive phenotype, but not directly to IPMN grading. They also detected the coexistence of ApoAII truncation and GNAS mutation in invasive cancer derived from IPMN [26].

A study on benign pancreatic lesions points out that there is no significant difference in the ApoAII modification level between low-risk IPMN patients and healthy controls. This finding suggests that the abnormal modification of ApoAII may not be a common feature of IPMN, but is closely related to the malignant transformation process. In the early stage of the tumor or low-grade dysplasia, carboxypeptidase-mediated C-terminal cleavage has not reached the detectable threshold. Only when the lesion progresses to high-grade dysplasia or invasive cancer, the truncated subtypes of ApoAII (such as the reduction of ApoAII-2 and the enrichment of ApoAII-4/5) show a significant imbalance [24,26]. So ApoAII is more suitable as a dynamic monitoring marker for malignant progression. The clinical application of this marker should focus on the risk stratification of high-risk IPMN and early prediction of malignant transformation, rather than on distinguishing benign from low-risk lesions.

Compared with other types of biomarkers, ApoAII is relatively mature and has been approved for commercialization in Japan. Its corresponding *in vitro* diagnostic (IVD) kit has been officially launched and is included in Japan’s medical insurance coverage. The Japan Pancreas Society has confirmed that ApoAII detection is covered by medical insurance, and combined testing of ApoAII and CA19-9 is available for routine clinical use [29,30]. Nevertheless, mature and low-cost ApoAII-specific kits dedicated to pancreatic cancer and IPMN are still unavailable in other regions, and their current application is largely limited to scientific research. Promoting the global clinical popularization and application of ApoAII remains an important research direction at present.

### 3.2 Inflammatory Markers

Inflammatory markers in the blood can be used as non-invasive and convenient tools for risk stratification. When combined with imaging features and tumor markers, they can improve the accuracy of predicting the malignant risk of IPMN. In IPMN, interleukin-18 (IL-18) initiates the inflammatory response and induces high expression of CCL19 [31]. The secreted CCL19 acts as a downstream effector to activate the JAK/STAT3 pathway, and IL-18 can independently stimulate this pathway as well, building a stable pro-inflammatory signaling loop [32]. This activated molecular axis profoundly disturbs immune homeostasis and promotes malignant phenotypic transformation of lesion cells. Correspondingly, the altered immune and inflammatory status can be evaluated by peripheral inflammatory biomarkers including NLR and SII, while the malignant transformation of epithelial cells is characterized by the upregulation of CEA.

Over recent years, many studies on inflammatory markers for IPMN risk stratification have been carried out. Among them, the neutrophil-to-lymphocyte ratio (NLR) is the focus of research.

Neutrophils have two opposite effects in the tumor microenvironment: inhibiting and promoting tumors. As NLR can be easily calculated from routine blood tests and is easily accessible, it has become a potential prognostic biomarker for many cancers. Over the past decade, many retrospective analyses have shown that NLR is an effective marker for evaluating the invasiveness of IPMN and the recurrence-free survival of patients with invasive IPMN. Combining NLR with imaging markers or traditional markers can effectively improve the detection efficiency and accuracy. The imaging markers include main pancreatic duct dilation, cyst size, enhancing solid components of the pancreatic cyst wall and the presence of mural nodules. The traditional markers include CEA and CA19-9 [33,34].

In 2017, Gemenetzis and others built a predictive nomogram. It combines NLR > 4, jaundice, main pancreatic duct dilation > 5 mm, cyst > 3 cm and enhancing solid components of the pancreatic cyst wall. This nomogram can accurately quantify the malignant risk of IPMN and can be used as an auxiliary tool for clinical decisions. The critical value of NLR varies across studies (2.0–4.0), which may be related to the scope of the analyzed samples [35]. In 2021, Kim et al. [36] proposed to set the critical value of NLR at 3.5 to ensure clinical practicability. At present, the predictive effect of NLR as a single factor is relatively low in all studies and needs to be improved. Moreover, NLR cannot distinguish between low and high-grade dysplasia of IPMN, and can only predict invasive cancer.

Besides NLR, other inflammatory markers are also mentioned in many studies, such as the platelet-to-lymphocyte ratio (PLR) and the advanced lung cancer inflammation index (ALI). Many studies show that PLR has no clinical value in evaluating different grades of IPMN [34,36]. Recently, Hata and others conducted a retrospective analysis. It included 171 IPMN patients who received surgical resection at Tohoku University Hospital from 2006 to 2018. They measured indicators such as Systemic Immune-inflammation Index (SII), Prognostic Nutritional Index (PNI), Maximum Standardized Uptake Value (SUVmax) and CA19-9, and conducted statistical analysis. Finally, they validated SII as a new biomarker for predicting high-risk IPMN. They also established a combined evaluation system integrating SII, SUVmax, CEA and imaging HRS. Its AUC reaches 0.824, with a sensitivity of 75.9%, a specificity of 80.0% and an accuracy of 77.2%. It significantly improves the accuracy of preoperative prediction of HGD/INV in IPMN [37].

In another single-center retrospective cohort study in 2022, researchers conducted univariate/multivariate regression analysis and Kaplan-Meier curve analysis on 76 pathologically confirmed IPMN patients. They found that C-reactive Protein/Albumin Ratio (CAR) is positively correlated with the malignant degree of IPMN. The Receiver Operating Characteristic (ROC) curve determined the optimal critical value of CAR as 0.011 (hs-CRP: mg/L, albumin: g/dL). High CAR is an independent predictor of IPMN malignant transformation and is related to patient prognosis. The AUC of high CAR alone for diagnosing HGD/INV is only 0.64, but it rises to 0.84 when combined with the MPD 5–9 mm indicator [38].

Inflammatory markers are valuable auxiliary tools for IPMN risk stratification. They have unique advantages especially in supplementing evaluation assessment and dynamically monitoring disease progression. But the majority of current studies are retrospective analyses, lacking multicenter prospective studies. Moreover, due to limitations such as insufficient specificity and the lack of unified standards, they cannot be used alone as the basis for IPMN risk stratification at present. They need to be combined with other examination methods to improve the accuracy of risk stratification and provide more reliable references for clinical decisions [1,39].

### 3.3 Telomere and Nucleic Acid Markers

#### 3.3.1 Cell-Free Circulating DNA (cfDNA)

Previous studies have exhibited that GNAS and KRAS are hotspot mutations in IPMN, and they are both important markers for risk stratification [40]. The latest genomic markers for IPMN risk stratification mainly focus on cell-free circulating DNA (cfDNA). cfDNA is free DNA in the blood, which can come from tumor or normal cells. As tumor-derived cfDNA, circulating tumor DNA (ctDNA) can be isolated but cannot be cultured *in vitro*. Only DNA-level information can be analyzed.

Detecting cfDNA in the blood is an important liquid biopsy method. It initially realized the early screening of cancers such as colorectal cancer and nasopharyngeal cancer [41]. Previous studies have verified the role of cfDNA in the identification and diagnosis of IPMN. A retrospective study used Droplet digital PCR (ddPCR) to focus on the IPMN hot spot mutations GNAS/KRAS. It verified the consistency of GNAS mutations between tissue and blood cfDNA in IPMN patients and the diagnostic effect of cfDNA for IPMN [42]. Another previous multicenter study used pre-amplification Digital Polymerase Chain Reaction (dPCR) technology to confirm the effectiveness of cfDNA in the identification of IPMN. It can efficiently detect KRAS/GNAS mutations in plasma cfDNA of patients with early resectable PDA and high-risk IPMN [43].

In recent years, the application of cfDNA in IPMN risk stratification has been continuously explored. A recent study compared the efficiency of cfDNA mutation detection in pancreatic juice and blood. Finally, it confirmed that there is no significant difference in the gene mutation detection rate between the two [44]. Building on previous research, another prospective study confirmed that IPMN, as precancerous lesions, can release cfDNA containing KRAS/GNAS mutations into the blood. Meanwhile, the researchers also explored the potential of relevant biomarkers for risk stratification of IPMN, including total plasma cfDNA concentration, hotspot mutations of KRAS and GNAS, and circulating epithelial cells. Ultimately, GNAS R201C (Sensitivity: 32.0%, Specificity: 95.7%), KRAS G12/A/C/D/R/S/V and G13D (Sensitivity: 16.0%, Specificity: 95.7%), as well as their combined panel (Sensitivity: 40.0%, Specificity: 95.7%) were identified as promising candidates. This finding provides a basis for molecular-level risk assessment and further reveals that it has high specificity for the detection of high-risk IPMN. It can be used as a reliable basis for conservative monitoring of branch duct-type IPMN without suspicious features [45]. Recently, Hartwig and his colleagues developed a methylated cfDNA biomarker panel based on non-invasive liquid biopsy. This signature panel consists of the top 50 differentially methylated Cytosine-phosphate-Guanine (CpG) loci screened via machine learning. Validated in the training cohort, the hybridization capture sequencing panel targeting circulating cell-free DNA methylation, when combined with the canonical clinical biomarker CA19-9, enables non-invasive diagnosis of pancreato-biliary malignancies and demonstrates robust translational feasibility. Subsequent analyses in an independent validation cohort further revealed that this assay effectively distinguish patients with pancreatobiliary cancer and HGD-IPMN from those with LGD-IPMN, pancreatitis, and healthy individuals, with an AUC of 0.88, a sensitivity of 92% and a specificity of 84%. It is significantly higher than the traditional CA19-9 test [46].

#### 3.3.2 miRNA

miRNAs are non-coding RNAs. They regulate the expression of target genes at the post-transcriptional level and participate in cancer occurrence and development. They are important oncogenes or tumor suppressor genes [47]. More and more studies focus on using miRNAs in the early diagnosis of pancreatic cancer. A 2015 genome-wide analysis study of IPMN screened six core differential miRNAs that are significantly downregulated in high-risk IPMN. They are miR-100, miR-99b, miR-99a, miR-342-3p, miR-126 and miR-130a. They have high efficiency in distinguishing high-risk IPMN. The AUC of the combination of miR-99b, miR-130a and miR-342-3p is 74%. It rises to 81% when combined with the status of main pancreatic duct involvement. Experimental verification shows that these miRNAs mainly regulate pathways related to tumor progression. The pathways include histone deacetylase, Hypoxia-Inducible Factor (HIF) hypoxia regulation, Vascular Endothelial Growth Factor (VEGF) signaling, Protein Kinase B (AKT) signaling and cytoskeleton remodeling. Their target genes are highly related to pancreatic tumors (*p* < 10^−29^) [48].

A subsequent 2016 study combined CT radiomic features with a plasma miRNA genomic classifier (MGC). It developed an innovative combined diagnostic model, which significantly improved the diagnostic effect for high-risk IPMN. The AUC of radiomics + First Principal Component + MGC is 0.92, which is better than single radiomics or MGC [49].

With further exploration, more efficient miRNA markers have been found. A 2020 study explored the potential of EV-miRNAs as screening and diagnostic biomarkers for IPMN and their malignant variant (IPMC). The screened EV-miR-4539 (AUC = 0.72) and EV-miR-6132 (AUC = 0.77) have high potential for screening IPMN in the general population and identifying IPMN with high malignant potential respectively [50]. Another study in the same year focused on the circulating exosomal miRNA characteristics of pancreatic and peripancreatic tumors. It clarified their advantages as markers for the specific identification of different pancreatic and peripancreatic lesions [51].

Recently, Zhang et al. [52] screened protein biomarkers and miRNA markers through high-throughput molecular analysis of serum proteins and PCR arrays. They obtained a miRNA marker group consisting of three core miRNAs: miR-122-5p, miR-125b-5p and miR-146a-5p. Existing protein and microRNA signatures present a perfect discriminatory ability (AUC = 1.00) to separate IPMN patients from healthy volunteers across training and independent validation cohorts. Such excellent performance is reasonable given the substantial biological difference between patients and healthy controls. For the more challenging clinical goal of stratifying low-risk and high-risk IPMN, a combined panel of five proteins and three microRNAs was developed, with an AUC of 0.97 in the independent validation cohort.

Similar to inflammatory markers, circulating miRNA panels also face identical challenges in clinical translation. Although multiple combined miRNA signatures have shown excellent diagnostic efficacy in distinguishing high-risk from low-grade IPMN, there remains a lack of unified cutoff thresholds for relative expression levels and standardized technical protocols. Variations in specimen pretreatment, RNA extraction methods, detection platforms and analytical pipelines across studies lead to poor repeatability and comparability of results. Most current investigations are limited to small-sample, single-center retrospective analyses, lacking large-scale multicenter prospective validation and unified quality control criteria. Additionally, peripheral blood miRNAs are vulnerable to interference by systemic inflammation, metabolic disorders and other malignancies, restricting their diagnostic specificity. At present, miRNA biomarkers are still confined to fundamental research and have not been incorporated into routine clinical grading systems or guideline recommendations [17,52,53]. Future efforts should prioritize standardizing procedures for specimen collection, exosome isolation and quantitative detection, establishing globally recognized cutoffs for core miRNA panels. Conducting external multicenter validation will help screen subtype-specific and stably expressed miRNA molecules. Furthermore, constructing multidimensional predictive models integrating miRNA signatures with imaging characteristics, inflammatory indicators and serum proteins is essential to accelerate the development of supporting detection kits and promote the IVD registration and clinical application of miRNA markers for IPMN.

#### 3.3.3 Telomere

Telomeres are specialized DNA-protein protective complexes located at the chromosomal ends of eukaryotic cells, functioning like a “protective cap” for chromosomes. They consist of highly repetitive TTAGGG DNA sequences combined with specific binding proteins. Early studies have confirmed the correlation between telomere alterations and the progression of IPMN. A study on genetic biomarkers in IPMN cyst fluid indicated that progressive severe telomere shortening occurs at the early stage of IPMN malignant transformation [54]. A retrospective single-center study published in 2008 enrolled 68 IPMN patients. Through Quantitative fluorescence *in situ* hybridization (Q-FISH) and immunohistochemical analysis of lesion tissues, researchers identified marked telomere shortening in early IPMN lesions accompanied by high Human telomerase reverse transcriptase (hTERT) expression, providing fundamental insights for subsequent investigations. They also investigated the positive rates of hTERT expression across all subtypes of IPMN and pancreatic ductal adenocarcinoma (PDAC), as well as the statistical differences between groups. The results were as follows: adenoma IPMN (15.8%), borderline IPMN (35.7%), carcinoma *in situ* IPMN (85.0%), invasive IPMN (86.7%), and PDAC (93.3%). hTERT exhibited favorable discriminatory performance for HGD-IPMN versus LGD-IPMN, indicating that hTERT can serve as a biomarker for risk stratification of IPMN [55].

In subsequent research, telomeres have become one of the research hotspots for IPMN risk stratification. Emerging evidence suggests that peripheral blood leukocyte telomere length can serve as a potential non-invasive biomarker for predicting IPMN malignant transformation. Furthermore, single-nucleotide polymorphisms (SNP) that determine leukocyte telomere length can assist in radiological risk stratification of IPMN. One study identified a telomere length-regulating single nucleotide polymorphism (SNP), PXK-rs6772228-A, which harbors predictive value for malignant progression risk of intraductal papillary mucinous neoplasms (IPMNs). In the discovery cohort, carriers of the A allele exhibited a hazard ratio (HR) of 3.17 for IPMN malignant transformation (95% confidence interval [CI]: 1.47–6.84, *p* = 3.24 × 10^−3^). A consistent risk tendency was observed in the validation cohort, yet the association failed to reach statistical significance [56]. In recent years, a large case–control study analyzing peripheral blood leukocyte telomere length in 1460 PDAC patients and 1459 healthy controls demonstrated that shortened leukocyte telomere length in treatment-naive newly diagnosed patients was significantly correlated with elevated PDAC risk. This finding laid a foundation for exploring the association between leukocyte telomere length and premalignant pancreatic lesions [57]. Subsequently, an Italian research team conducted a prospective observational study including 361 IPMN patients, establishing a teloscore based on 11 telomere-related Single Nucleotide Polymorphisms (SNPs) to explore the relationship between leukocyte telomere length and the occurrence of worrisome features, high-risk stigmata, and malignant progression in IPMN. Consistent with previous research findings, the A allele of PXK-rs6772228 shortens leukocyte telomere length and accelerates the malignant progression of intraductal papillary mucinous neoplasms (IPMNs) toward pancreatic ductal adenocarcinoma, and can serve as a germline genetic biomarker for risk stratification in patients with IPMNs. Nevertheless, this study failed to achieve statistically significant results regarding the gene’s predictive value for the long-term malignant transformation risk of IPMNs in the validation cohort [58].

Nevertheless, leukocyte telomere length only reflects the systemic level of telomere attrition, whereas early malignant transformation of IPMN is characterized by localized progressive telomere shortening and fusion. This discrepancy makes leukocyte telomere length unable to precisely mirror the malignant degree of local lesions. In addition, leukocyte telomere length is strongly affected by multiple confounding factors, resulting in insufficient diagnostic specificity of telomere-related indicators. From the perspective of clinical translation, existing studies are mainly limited to Caucasian populations in Italy, lacking validation from large-sample multicenter prospective cohorts [57,58]. Moreover, telomere detection is costly with narrow applicable scenarios; compared with CA19-9 and radiological examinations, its clinical benefit remains unclear, limiting widespread clinical popularization. Future research should focus on further external validation, supplementing long-term follow-up evidence, establishing unified standardized detection protocols, and optimizing detection costs to promote the clinical application of telomere-related biomarkers in IPMN grading.

## 4 Clinical Translation: New Achievements and Dilemmas

### 4.1 Current Clinical Translation Achievements

Currently, the core blood marker endorsed by clinical guidelines remains the traditional CA19-9. However, in recent years, a growing body of clinical translation achievements has offered many new alternatives for non-invasive IPMN risk stratification using blood markers in clinical settings. Recently, Inflammatory indicators such as NLR feature easy accessibility, low cost and high detection efficiency. Compared with other biomarkers, they have been extensively and deeply investigated, representing a relatively mature category of non-invasive blood markers for IPMN grading. Gradually, they have been integrated into routine clinical practice, serving as a powerful adjunct to assist surgical decision-making and optimize traditional predictive models [36]. Nevertheless, similar to conventional biomarkers, inflammatory indicators are generally only assigned auxiliary weighting values and cannot independently determine surgical indications.

In 2024, ApoAII was approved in Japan as an *in vitro* diagnostic (IVD) kit for the auxiliary diagnosis of pancreatic cancer and incorporated into public medical insurance schemes. Studies have demonstrated that its effect in IPMN risk stratification is better than the traditional marker CA19-9, and it is currently being gradually applied in clinical practice [59]. The Japanese Pancreatic Society recommends combined detection based on CA19-9 as a strategy for IPMN risk stratification. In some other regions, ApoAII has also undergone gradual commercialization; however, its application is largely confined to research settings, and it cannot be adopted for clinical IPMN risk stratification or surgical decision guidance [29,30].

miRNA panel markers have exhibited extremely high diagnostic effect in multicenter studies (AUC up to 0.97) [52]. cfDNA enables the detection of characteristic mutations of IPMN, offering a novel approach for non-invasive molecular stratification. It is still in the verification stage and has high potential [45]. Other potential biomarkers, including MUC5AC identified in blood extracellular vesicles (EVs) that are currently in the early research phase, hold promise as novel markers for IPMN risk stratification in future investigations [11].

Beyond preoperative risk assessment, studies have also indicated that blood-based biomarkers possess substantial potential for assessing patient prognosis and guiding postoperative management. For instance, preoperative indicators such as NLR, platelet-to-lymphocyte ratio (PLR), and CA19-9 can be utilized to predict the risk of postoperative recurrence and survival outcomes in IPMN patients. Dynamic monitoring of postoperatively changing biomarkers facilitates the early detection of recurrence and timely intervention [17].

The clinical translation of blood biomarkers has broken through the limitation of single preoperative risk stratification and covered multiple clinical diagnosis and treatment scenarios. These markers are suitable for non-invasive primary screening of asymptomatic pancreatic cystic lesions in outpatient settings and may help identify patients who are most likely to require endoscopic ultrasonography (EUS) and invasive puncture procedures [52]. Moreover, they are suitable for the long-term follow-up and dynamic monitoring of low-risk branch duct-intraductal papillary mucinous neoplasm (BD-IPMN), which reduces repeated enhanced CT/MRI examinations and lowers radiation exposure as well as medical costs [60]. Additionally, these biomarkers can support early warning of postoperative recurrence and prognostic stratification of IPMN patients, enabling the establishment of individualized postoperative follow-up frequency regimens [8]. Meanwhile, they can be incorporated into the preliminary early screening system for pancreatic cancer in high-risk populations, including the elderly population, patients with new-onset diabetes, and individuals with a family history of pancreatic neoplasms, facilitating the early identification and intervention of precancerous lesions [61].

Nevertheless, it is noteworthy that the latest IAP Kyoto Guidelines and ESMO Guidelines only regard novel blood biomarkers as potential research indicators and have not incorporated them into routine recommended grading metrics. The core reasons lie in the lack of large-sample population cutoff values and the absence of unified standardized detection paradigms, which constitute the key bottleneck restricting further clinical translation and routine clinical application of these biomarkers [8].

### 4.2 Dilemmas in Clinical Translation

Currently, the core challenge associated with blood-based markers lies in the fact that no single marker can independently, accurately, and standardly accomplish IPMN risk stratification. Most blood markers fail to balance specificity and sensitivity. For example, while inflammatory indicators exhibit high sensitivity, they are prone to interference from a variety of conditions, including most tumors, inflammatory processes, and systemic infections, lacking clinical specificity and thus serving only as a weak adjunct to imaging examinations. Furthermore, they are often unable to distinguish IPMN subtypes with low malignant potential, such as branch duct-type IPMN, small cysts, and those without mural nodules—precisely the subtypes that pose the greatest challenges in clinical decision-making and require rigorous risk stratification.

Meanwhile, a unified detection platform, standardized cutoff values, and standardized quality control represent the primary barrier to the clinical translation of most blood-based biomarkers. Current guidelines still regard blood markers as auxiliary tools to supplement imaging, and they cannot be used alone as the basis for surgical or follow-up decisions [52,62]. At present, a large number of biomarkers, including NLR, cutoff values of ApoAII subtypes, and relative expression levels of miRNAs, lack unified optimal threshold criteria. Detection platforms adopted in different studies are incompatible with one another, and no standardized protocols have been established for specimen preprocessing. These deficiencies fail to meet the requirements for *in vitro* diagnostic (IVD) registration and routine clinical access, constituting a major obstacle restricting the clinical translation of blood biomarkers for IPMN risk stratification.

On the other hand, blood-based biomarkers for IPMN risk stratification face inherent bottlenecks at the molecular biological level. IPMN lesions have extremely high heterogeneity. Local molecular changes in pancreatic cysts (such as GNAS/KRAS mutations and abnormal mucin) are difficult to release into the peripheral blood, resulting in weak blood signals and low detection rates. Additionally, the elevation of blood biomarkers typically lags behind histological malignant transformation, limiting their value in early warning and making it challenging to achieve “early intervention” [62]. Most importantly, current biomarkers are mostly pan-pancreatic cancer markers, and there is no truly specific target for IPMN malignant transformation. As a result, it remains difficult to accurately distinguish the progression of precancerous lesions from ordinary inflammation or benign hyperplasia.

From a clinical perspective, surgical decision-making mainly relies on imaging examinations and cytological/histological biopsies, such as endoscopic ultrasound-guided fine-needle aspiration (EUS-FNA). in accordance with major clinical guidelines, while blood biomarkers can only serve as auxiliary indicators. Meanwhile, previous medical disputes caused by overtreatment with unnecessary surgery and missed diagnoses have made clinicians more conservative in decision-making, rendering them reluctant to adopt novel non-invasive biomarkers. On the other hand, certain biomarkers such as cfDNA and DEST-EV incur relatively high detection costs and are not covered by medical insurance, limiting their application to special medical needs and academic research only. Furthermore, primary medical institutions lack professional testing equipment and are unable to carry out PCR, ELISA and sequencing assays, thereby forming a clear gap in medical service capacity across different medical centers.

### 4.3 The Future Clinical Implementation Pathway

In view of the current dilemmas existing in clinical translation, medical researchers can make efforts from multiple aspects. First, for those biomarkers with insufficient research progress and unavailable clinical application, investigators should actively carry out multicenter prospective cohort studies to establish stratified cutoff values for IPMN, especially for BD-IPMN. Meanwhile, the development of low-cost integrated detection kits, as well as the simplification of DEST and cfDNA detection workflows, is also an essential part of clinical translation, which can reduce clinical application costs and lower the technical threshold for biomarker detection.

For relatively mature biomarkers, it is necessary to construct a full-course application pathway of non-invasive biomarkers covering high-risk population screening, outpatient preliminary screening, preoperative risk stratification, and postoperative surveillance. By unifying standardized protocols for specimen collection, critical cutoff values, sample preprocessing and detection platforms, researchers should promote the establishment of a global standardized quality control system for novel blood biomarkers of IPMN.

## 5 Limitations and Future Directions

In recent years, research on non-invasive grading blood markers for IPMN has achieved multi-dimensional breakthroughs. Research has shifted from exploring traditional single markers to adopting multi-marker models, including serum proteins, inflammatory indicators, telomeres and nucleic acids. Meanwhile, combined with innovations in multi-omics technology and liquid biopsy technology, the accuracy and specificity of non-invasive IPMN grading have been significantly improved, providing a new strategy to address the limitations of the traditional clinical grading system [52].

**Figure 3:**
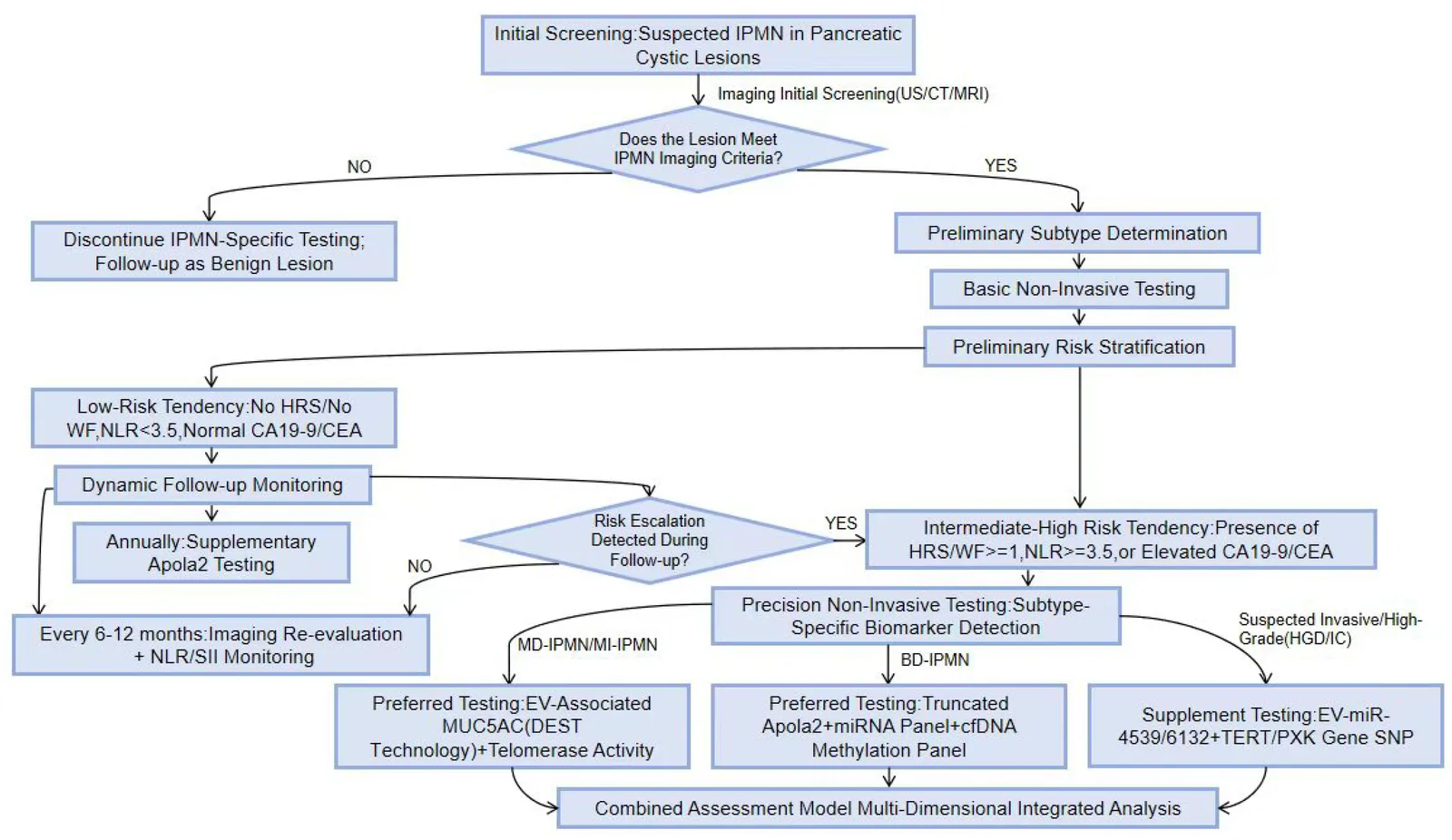
This proposed flowchart illustrates the non-invasive screening and risk stratification pathway for suspected intraductal papillary mucinous neoplasms (IPMN). Initial imaging (US/CT/MRI) confirms IPMN imaging criteria; non-conforming lesions are followed as benign. Confirmed cases undergo preliminary subtype determination, basic non-invasive testing, and risk stratification. Low-risk cases (no high-risk stigmata/worrisome features, NLR < 3.5, normal CA19-9/CEA) receive dynamic follow-up (annual supplementary ApoA2 testing, imaging/NLR/SII monitoring every 6–12 months). For risk escalation or intermediate-high risk cases (≥1 high-risk stigmata/worrisome features, NLR ≥ 3.5, elevated CA19-9/CEA), subtype-specific precision testing is initiated: MD-IPMN/MI-IPMN use EV-associated MUC5AC (DEST technology) + telomerase activity; BD-IPMN use truncated ApoA2 + miRNA panel + cfDNA methylation panel. Suspected invasive/high-grade lesions receive supplementary EV-miR-4539/6132 + TERT/PXK gene SNP testing. A multi-dimensional integrated analysis via a combined assessment model guides clinical decision-making.

Each type of marker has its unique advantages. Serum protein markers such as ApoAII and EV-related MUC5AC exhibit the potential for disease specificity, and can accurately target the key stages of IPMN malignant transformation [11,23]. Inflammatory indicators have become routine clinical auxiliary tools due to their accessibility and low cost, fulfilling the demand for non-invasive detection in primary diagnosis and treatment [34]. Telomere and nucleic acid markers reveal the molecular mechanisms underlying IPMN malignant progression from a molecular perspective, enabling a leap from “phenotypic assessment” to “molecular stratification”. The diagnostic efficacy of the combined detection of multiple markers is of greater clinical value, which is worthy of further exploration [63]. We summarized all hot biomarkers and biomarker combinations included in this review in Table 1 and Table 2, and presented the preliminarily sorted clinical application pathway in Fig. 3.

**Table 1:** Table of sorted single biomarkers.

Type	Biomarker	Discrimination Target	Diagnostic Performance	Clinical Application	Recommendation Grade	Reference
EVs	MUC5AC	IC vs. LGD-IPMN	Sensitivity: 100%, Specificity: 82%, Accuracy 96%, AUC = 1.0 (Data were derived from the discovery cohort and validation cohort.)	Used alone, it accurately distinguishes invasive IPMN for surgical triage.	high	[11]
	GPC1	IC vs. LGD-IPMN	Sensitivity: 73%, Specificity: 98%	Has limited discriminatory value alone; its expression in high-grade IPMN is slightly lower than that in low-grade IPMN	low	[11]
	TSP1	IC vs. LGD-IPMN	Sensitivity: 64%, Specificity: 93%	The overall signal is close to the background with large overlap between groups, resulting in low clinical application value alone	low	[11]
ApoAII	ApoAII-i index	Healthy subjects/LGD-IPMN vs. IPMN with HRS/WF	For distinguishing HRS + WF from low-risk cases: AUC = 0.676; outstanding discriminatory ability for cases with cyst ≥ 30 mm, MPD ≥ 5 mm, and positive mural nodule	Can be directly matched with the imaging stratification criteria of HRS/WF in the Kyoto Guidelines; can be used as serological supplementary evidence for imaging high-risk stigmata, and is more suitable for routine clinical screening and clinical translation of diagnostic kits	high	[23]
Inflammatory Markers	NLR	IC/HGD-IPMN vs. LGD-IPMN, IC-IPMN vs. HGD/LGD-IPMN	IC/HGD-IPMN vs. LGD-IPMN AUC = 0.605–0.799, IC-IPMN vs. HGD/LGD-IPMN AUC = 0.62–0.836 Patients with high NLR have significantly shortened DFS	Can independently predict the invasion risk of IPMN alone, with specificity superior to sensitivity. It is difficult to distinguish LGD from HGD alone. It is affected by systemic infection, biliary tract inflammation, and preoperative invasive procedures, resulting in false positives	medium	[33,34,35,36]
	PLR	Non-invasive IPMN vs. Invasive IPMN	PLR in the invasive group is significantly higher than that in the non-invasive group (*p* < 0.05); its diagnostic performance is weaker than NLR	Has limited predictive value alone, and is mostly used as an auxiliary reference; no significant incremental benefit when combined with imaging, and NLR is clinically preferred	low	[34,36]
	SII	IC/HGD-IPMN vs. LGD-IPMN	Sensitivity: 38.8%, Specificity: 87.3%, AUC = 0.628	Compared with single markers such as CA19-9, CEA, PNI and NLR, SII can distinguish LGD from simple HGD, making up for the shortcoming that NLR can only identify invasive carcinoma. However, it lacks sufficient sensitivity, so its separate use for screening tends to miss high-risk IPMN.	medium	[37]
	CAR	IC/HGD-IPMN vs. LGD-IPMN	Sensitivity: 38.8%, Specificity: 87.3%, AUC = 0.602	Compared with NLR, it can earlier indicate high-grade dysplasia, which compensates for the limitation of NLR that can only detect invasion; high CAR indicates shortened postoperative disease-free survival, and has both diagnostic and prognostic value	medium	[38]
cfDNA	GNAS R201C	IC/HGD-IPMN vs. LGD-IPMN	Sensitivity: 32.0%, Specificity: 95.7%	Can be used for risk stratification of IPMN, The presence of mutation can indicate intensified imaging monitoring	medium	[45]
	KRAS hotspot mutations (G12/A/C/D/R/S/V/G13D)	IC/HGD-IPMN vs. LGD-IPMN	Sensitivity 16%, Specificity: 95.7%	The positive rate increased significantly only in branch duct IPMN with suspicious signs (*p* = 0.043), and its independent diagnostic value was inferior to that of GNAS	medium	[45]
miRNA	EV-miR-4539	IPMN vs. non-tumor healthy controls	AUC = 0.72, Sensitivity 60.5%, Specificity: 95.2%	Applicable for non-invasive population-level screening of IPMN; convenient and repeatable	medium	[50]
	EV-miR-6132	IPMN-derived carcinoma (IPMC) vs. benign IPMN	AUC = 0.77, Sensitivity 88.3%, Specificity: 65.4%	Identifies high malignant potential IPMN; expression unaffected by biliary obstruction, overcoming reduced accuracy of CA19-9 in obstructed patients; aids clinical timing of surgery	high	[50]
Telomere	LTL	IPMN malignant progression	LTL shortening is an early event in IPMN malignant transformation; progressive shortening from adenoma to carcinoma *in situ*, with no further shortening thereafter; distinguishes IPMN pathological grades.	Used for IPMN risk stratification and prediction of progression to PDAC; minimally invasive peripheral blood test, repeatable, suitable for clinical screening and dynamic monitoring	medium	[56,57,58]
	hTERT	IC/HGD-IPMN/PDAC vs. LGD-IPMN	Accuracy: adenoma IPMN 15.8%, borderline IPMN 35.7%, carcinoma *in situ* IPMN 85.0%, invasive IPMN 86.7%, PDAC 93.3%. significantly higher positivity in carcinoma *in situ* and above vs. borderline and below (*p* < 0.005)	Marker of IPMN malignant progression; identifies high-risk IPMN subgroups to guide surgery; detectable by IHC, simple and low-cost for routine clinical pathology	medium	[55]
	PXK-rs6772228-A	Long-term risk of IPMN progressing to invasive pancreatic cancer	HR = 3.17, 95%CI: 1.47–6.84, *p* = 0.003; adjusted for cyst diameter: HR = 2.43, 95%CI: 1.11–5.32, *p* = 0.026; significant association in combined cohort (*p* = 0.012)	Genetic marker for IPMN progression risk. But it is only used to predict the long-term risk of IPMN progressing to invasive carcinoma, and is not an immediate lesion grading marker	low	[56,58]

EVs, Extracellular Vesicles; GPC1, Glypican 1; TSP1, Thrombospondin 1; ApoAII, Apolipoprotein A-II; HRS, High-risk stigmata; WF. Worrisome Features; AUC, Area Under the ROC Curve; IPMN, Intraductal Papillary Mucinous Neoplasm; HGD, High-grade Dysplasia; MGD, Moderate-grade Dysplasia; LGD-IPMN, Low-grade Dysplasia; IC, Invasive Carcinoma; PDAC, Pancreatic Ductal Adenocarcinoma; CAR, Carcinoembryonic Antigen Ratio; NLR, Neutrophil-to-Lymphocyte Ratio; PLR, Platelet-to-Lymphocyte Ratio; SII, Systemic Immune-Inflammation Index; PNI, Prognostic Nutritional Index; CA19-9, Carbohydrate Antigen 19-9; CEA, Carcinoembryonic Antigen; hTERT, Human Telomerase Reverse Transcriptase; HR, Hazard Ratio; DFS, disease-free survival; MPD, main pancreatic duct; LTL, Leukocyte telomere length; IHC, Immunohistochemistry.

Nevertheless, there are still many pressing issues in this research field, which also serve as key bottlenecks restricting the clinical translation of blood markers. On the one hand, the specificity and application scope of markers are limited. Most inflammatory indicators are easily interfered by systemic inflammation and other tumors. Existing protein and nucleic acid markers are mostly universal targets for pancreatic cancer, lacking truly specific targets for IPMN malignant transformation. They cannot effectively distinguish the progression of IPMN precancerous lesions from benign pancreatic hyperplasia. Furthermore, it is difficult to achieve accurate stratification for the low-risk BD-IPMN subtype, which poses the greatest challenge in clinical decision-making [64]. On the other hand, the detection system lacks standardization. There are significant differences in the cut-off values and detection methods of markers across different studies. For instance, the cut-off value of NLR ranges from 2.0 to 4.0. Various approaches for miRNA extraction and isolation lead to systematic errors. Without a unified detection platform and quality control standards, the results are difficult to reuse in clinical practice [34].

In addition, the biological characteristics of IPMN lesions bring inherent obstacles. IPMN lesions have extremely high heterogeneity, and molecular changes in pancreatic cysts are difficult to release into the peripheral blood, resulting in weak tumor-related signals and low detection rates in the blood. Most blood markers are elevated later than histological malignant transformation, so the early warning value is limited, and it is impossible to achieve the core clinical demand of “early screening and early intervention” for IPMN [65].

Another noteworthy aspect is that the clinical application status of these markers remains at the auxiliary level. Existing guidelines still regard blood markers as a supplement to imaging assessment. There is insufficient evidence from multicenter prospective studies to support their application as an independent basis for surgical or follow-up decisions. Most studies are retrospective, lacking long-term follow-up data to verify the dynamic monitoring value of markers [8,40].

Notably, the development of non-invasive IPMN grading needs to balance clinical practicality and technical accessibility. There are differences in the detection technology levels among different medical centers. For example, technologies such as DEST and cfDNA methylation detection yield excellent results but have high requirements for detection equipment and technical personnel, making them difficult to promote in primary medical institutions. In contrast, easily accessible markers such as inflammatory indicators and CA19-9 have limited efficacy. How to balance detection effectiveness and accessibility, and establish a hierarchical non-invasive detection system, has become an important practical consideration for clinical translation [11].

In the future, the research and clinical translation of non-invasive grading blood markers for IPMN should focus on four directions. First, it is extremely urgent to conduct multicenter, large-sample, and prospective cohort studies to explore and verify specific molecular targets for IPMN malignant transformation. We need to break through the limitations of universal pancreatic cancer markers and, in particular, develop exclusive stratification markers for the low-risk BD-IPMN subtype [52]. Second, there is an urgent need to advance the standardization and normalization of detection technologies by unifying the detection platforms, cutoff values, and quality control standards for various markers, so as to resolve the heterogeneity in results across different studies. Meanwhile, develop low-cost and easy-to-operate detection technologies to improve accessibility in primary medical institutions [11]. Third, constructing a multidimensional combined evaluation model that integrates blood markers, imaging features, clinicopathological parameters, and multi-omics data is the future direction. We should fully leverage the strengths of diverse detection approaches to achieve precise stratification and individualized diagnosis and treatment of IPMN [52]. Last but not least, the molecular mechanisms of IPMN malignant transformation and the correlation between markers and IPMN pathological grades and subtypes still need to be further explored theoretically. Future studies should investigate the early detection value of these markers and drive the transition of IPMN clinical management from “passive follow-up” to “active intervention” [8,65].

**Table 2:** Table of biomarker panel.

Type	Biomarker Panel	Discrimination Target	Diagnostic Performance	Clinical Application	Recommendation Grade	Reference
Traditional Biomarkers	CEA + CA19-9	Healthy subjects vs. all grades of IPMN	No unified threshold is set in the Kyoto Guidelines, but a large number of evidence-based studies have proven that the two have a complementary effect	The most important adjunct diagnostic and therapeutic tool, which must be used in stratified combination with imaging/EUS	medium	[8]
EVs	MUC1 + GPC1 + EGFR + EpCAM + WNT-2	IC-IPMN vs. LGD-IPMN	Sensitivity: 70%, Specificity: 68%, diagnostic accuracy up to 86%	The panel has high specificity but low sensitivity, which is inferior to the single MUC5AC biomarker	medium	[11]
	DEST-MUC5AC EV + imaging	IC-IPMN vs. LGD-IPMN	Sensitivity: 95%, Specificity: 100%; diagnostic accuracy up to 100%	The combination can compensate for the insufficient sensitivity of MUC5AC and the high missed diagnosis rate of imaging, and is recommended for exploratory clinical use	high	[11]
ApoAII	ApoAII-i + CA19-9	Healthy/LGD-IPMN vs. HGD-IPMN	The combined Specificity: 62.1%, and the Sensitivity: is increased from 69.6% (single biomarker) to 72.0%	The combination only yielded slight numerical improvements without statistically significant differences; CA19-9 confers almost no added value for risk stratification of IPMNs, so routine combined testing is not recommended in clinical practice.	low	[23]
Inflammatory Markers	NLR + CEA + CA19-9	LGD-IPMN, HGD-IPMN and IC	Sensitivity: 58.8% Specificity: 76.8%, Accuracy 64.9%, PPV 83.3%, which is higher than any single indicator	Serotonin and serum CA19-9 have low early sensitivity, which can be compensated by NLR	medium	[33]
	inflammatory markers (NLR/CAR) + imaging (CT/MRI/EUS)	IPMN risk stratification, surgical indication	The combined positive predictive value for malignant prediction is significantly increased, especially in patients with concerning imaging features	It is the mainstream first-line clinical combination regimen, which fits the process of the Kyoto Guidelines; it can further stratify cases in the imaging gray zone, and can be used for risk stratification before EUS puncture	high	[33,34,35,36]
	CEA + SII+ SUVmax + HRS	IC/HGD-IPMN vs. LGD-IPMN	AUC = 0.824, Sensitivity: 75.9% Specificity: 80.0%, Accuracy 77.2%	Compared with single diagnostic standards relying solely on inflammation indicators, tumor markers or imaging examinations, multi-dimensional combined testing simultaneously improves sensitivity and specificity, and its stratified accuracy is markedly superior to all single-index and dual-index combinations. Its drawback lies in its dependence on PET, making it difficult to popularize at primary medical institutions.	medium	[37]
cfDNA	50 Methylated CpG + CA19-9	PBC + HGD-IPMN vs. LGD-IPMN + Pancreatitis + Healthy Controls	validation cohort (*n* = 37): AUC = 0.88, Sensitivity: 92%, Specificity: 84%;	It can be used for non-invasive early diagnosis of pancreatic cancer and risk stratification of precancerous lesions (IPMN), with performance superior to the traditional biomarker CA19-9, and is suitable for routine clinical use	high	[46]
	KRAS + GNAS	HGD-IPMN vs. LGD-IPMN	Sensitivity: 40%, Specificity: 95.7%	It can be used for risk stratification of IPMN, distinguishing high-risk IPMN requiring surgical resection from low-risk IPMN suitable for conservative monitoring, and a negative result can basically indicate the absence of high-grade/invasive lesions	medium	[45]
miRNA	5-proteins (EEF1A1, RPH3AL, NCOR1, L1CAM, TMEM161A) + 3-miRNAs (miR-146a-5p, miR-155-5p, miR-375) panel	IC/HGD-IPMN vs. LGD-IPMN	training cohort AUC = 0.99, Independent validation cohort AUC = 0.97	It can be used for IPMN, especially for patients without high-risk radiological features, which can significantly improve the diagnostic accuracy, reduce unnecessary surgery and missed diagnosis. It only requires peripheral blood for detection, which is minimally invasive and convenient.	high	[52]
	3-miRNAs panel: miR-122-5p, miR-125b-5p, miR-146a-5p	HGD-IPMN vs. healthy subjects; LGD-IPMN vs. healthy subjects (additional combination with miR-375 required)	High-risk IPMN vs. healthy subjects: AUC = 1.00, Sensitivity: 100%, Specificity: 100%	It can be used for non-invasive early screening of IPMN. The core panel only contains 3 miRNAs, with convenient detection process, low cost and extremely high accuracy. It is suitable for promotion in routine clinical physical examination and can realize early identification of IPMN.	medium	[52]
	WF + radiomic + MGC	IC/HGD-IPMN vs. LGD-IPMN	AUC = 0.93, Sensitivity: 89%, Specificity: 89%, PPV: 89%, NPV: 89%	This triple-combination model integrates suspicious imaging signs, radiomics First Principal Component and plasma miRNA classifier MGC, with complementary information and balanced diagnostic performance. It can address the stratification challenge of IPMNs with only suspicious signs, being non-invasive and superior to single/two-biomarker models. However, limited by small-sample single-center retrospective data and the lack of unified standards for image acquisition and segmentation, external validation is still required prior to clinical application.	high	[49]

EVs, Extracellular Vesicles; GPC1, Glypican 1; MUC1, Mucin 1, cell surface associated; EGFR, Epidermal Growth Factor Receptor; EpCAM, Epithelial Cell Adhesion Molecule; WNT-2, Wnt family member 2; CA19-9, Carbohydrate Antigen 19-9; CEA, Carcinoembryonic Antigen; AUC, Area Under the ROC Curve; IPMN, Intraductal Papillary Mucinous Neoplasm; HGD, High-grade Dysplasia; MGD, Moderate-grade Dysplasia; LGD, Low-grade Dysplasia; IC, Invasive Carcinoma; PDAC, Pancreatic Ductal Adenocarcinoma; CAR, Carcinoembryonic Antigen Ratio; NLR, Neutrophil-to-Lymphocyte Ratio; PLR, Platelet-to-Lymphocyte Ratio; SII, Systemic Immune-Inflammation Index; HRS, High-risk stigmata; WF. Worrisome Features; MGC, miRNA genomic classifier (miR-200a-3p, miR-1185-5p, miR-33a-5p, miR-574-3p, miR-664b); PPV, Positive Predictive Value; PET, Positron Emission Tomography; PBC, Pancreato-Biliary Cancer; SUVmax, Maximum Standardized Uptake Value; NPV, Negative Predictive Value.

## 6 Conclusions

This review updates the latest advances in non-invasive blood biomarkers for grading IPMN, covering serum proteins, inflammatory indicators, telomere-related, and nucleic acid markers. In recent years, these biomarkers have gradually moved from bench to bedside. Molecules such as ApoAII, EV-associated MUC5AC, cfDNA methylation panels, miRNA signatures, and telomere-related markers show better diagnostic potential than conventional CA19-9 and CEA. The combination of multiple biomarkers and imaging remarkably improves the accuracy of IPMN risk stratification, reduces unnecessary surgery, and aids early pancreatic cancer screening, prognosis evaluation and postoperative monitoring. Easy-to-measure inflammatory indicators have also become routine auxiliary tools for non-invasive IPMN grading.

The main bottlenecks hinder the clinical translation of blood-based IPMN markers, including unsatisfactory specificity, limited application range, non-standard detection protocols and insufficient clinical practicability. On this basis, this review puts forward relevant suggestions for future research. With the progress of multi-omics, liquid biopsy and tumor microenvironment research, these non-invasive biomarkers are expected to serve as core tools for clinical risk stratification.

## Data Availability

Not applicable.
